# Transcriptional mechanism of vascular endothelial growth factor-induced expression of protein kinase CβII in chronic lymphocytic leukaemia cells

**DOI:** 10.1038/srep43228

**Published:** 2017-02-24

**Authors:** Ola Al-Sanabra, Andrew D. Duckworth, Mark A. Glenn, Benjamin R. B. Brown, Piera Angelillo, Kelvin Lee, John Herbert, Francesco Falciani, Nagesh Kalakonda, Joseph R. Slupsky

**Affiliations:** 1 Department of Molecular and Clinical Cancer Medicine, University of Liverpool. 1st Floor Sherrington Building, Ashton Street, Liverpool, L69 3GE, United Kingdom; 2 Department of Medicine, Roswell Park Cancer Center, Elm and Carlton St, Buffalo, NY, 14263, USA; 3 Technology Directorate Computational Biology Facility, University of Liverpool, Biosciences Building, Crown Street, Liverpool, L69 7ZB, United Kingdom; 4 Department of Functional and Comparative Genomics, University of Liverpool Biosciences, Building, Crown Street, Liverpool, L69 7ZB, United Kingdom

## Abstract

A key feature of chronic lymphocytic leukaemia (CLL) cells is overexpressed protein kinase CβII (PKCβII), an S/T kinase important in the pathogenesis of this and other B cell malignancies. The mechanisms contributing to enhanced transcription of the gene coding for PKCβII, *PRKCB*, in CLL cells remain poorly described, but could be important because of potential insight into how the phenotype of these cells is regulated. Here, we show that SP1 is the major driver of PKCβII expression in CLL cells where enhanced association of this transcription factor with the *PRKCB* promoter is likely because of the presence of histone marks permissive of gene activation. We also show how vascular endothelial growth factor (VEGF) regulates *PRKCB* promoter function in CLL cells, stimulating PKCβ gene transcription via increased association of SP1 and decreased association of STAT3. Taken together, these results are the first to demonstrate a clear role for SP1 in the up regulation of PKCβII expression in CLL cells, and the first to link SP1 with the pathogenesis of this and potentially other B cell malignancies where PKCβII is overexpressed.

Chronic lymphocytic leukaemia (CLL) is a common malignancy of mature B cells^1^,^2^. A distinctive feature of the malignant cells in this disease is overexpression of protein kinase CβII (PKCβII)^3^, a classical PKC isoform that is involved in a wide variety of cellular processes^4^. PKCβII is important to the pathophysiology of CLL cells because of its role in regulating B cell receptor (BCR) signalling^5^,^6^,^7^,^8^, and because it can enhance cell survival by activating Akt^9^ and phosphorylating Bcl2 at the mitochondrial membrane^10^. Finally, studies using the Tcl1 mouse model of CLL have shown that disease fails to develop when the gene encoding PKCβII, *PRKCB*, is genetically deleted^11^.

In addition to CLL, overexpression of PKCβII is also observed in B-lymphocyte malignancies such as diffuse large B cell and mantle cell lymphoma^12^,^13^, and in epithelial tumours such as carcinoma of the colon^14^ and breast^15^. Indeed, like CLL, the development of colon cancer is intrinsically linked with overexpressed PKCβII^16^,^17^. Therefore, understanding how expression of this protein is regulated may give insight into the pathogenesis and progression of CLL and other cancers.

The basal promoter region of *PRKCB* is characterised^18^,^19^ with early studies identifying binding sites for the transcription factors (TF) MITF^20^ and RUNX1^21^. Experiments in more recent literature have demonstrated additional binding sites for SP1^22^ as well as for STAT3^23^. However, how these TFs contribute to overexpression of *PRKCB* in the malignant cells of CLL and other cancers is poorly described. Potential insight into this mechanism is provided by previous work from this Department showing *PRKCB* transcription can be induced in CLL cells by VEGF-induced stimulation of PKCβII activity^24^. This mechanism is also reportedly used in other cell systems^25^,^26^, and may be of particular importance to the pathogenesis of CLL because of the high levels of this cytokine present within tissues where expansion of the malignant clone takes place^27^,^28^.

In the present study we show SP1 is a major driver of PKCβII overexpression in primary CLL cells. Enhanced gene transcription of *PRKCB* in CLL compared to normal B cells is likely the result of increased access of SP1 to the gene promoter region facilitated by the presence of permissive histone marks. We also find that STAT3 has a suppressive role for the activity of the *PRKCB* promoter in CLL cells and increased binding of STAT3 to this site is linked with decreased association of SP1. Treatment with VEGF causes a decrease in STAT3 binding to the *PRKCB* promoter and maintains elevated binding of SP1 during *in vitro* culture. Taken together, these results demonstrate a direct relationship between SP1 binding and *PRKCB* transcription, and further suggest that this TF is a contributor to the pathobiology of CLL and potentially other malignant cells where PKCβII is overexpressed.

## Results

### SP1 mediates PRKCB transcription in CLL and MEC1

**Figure 1:**
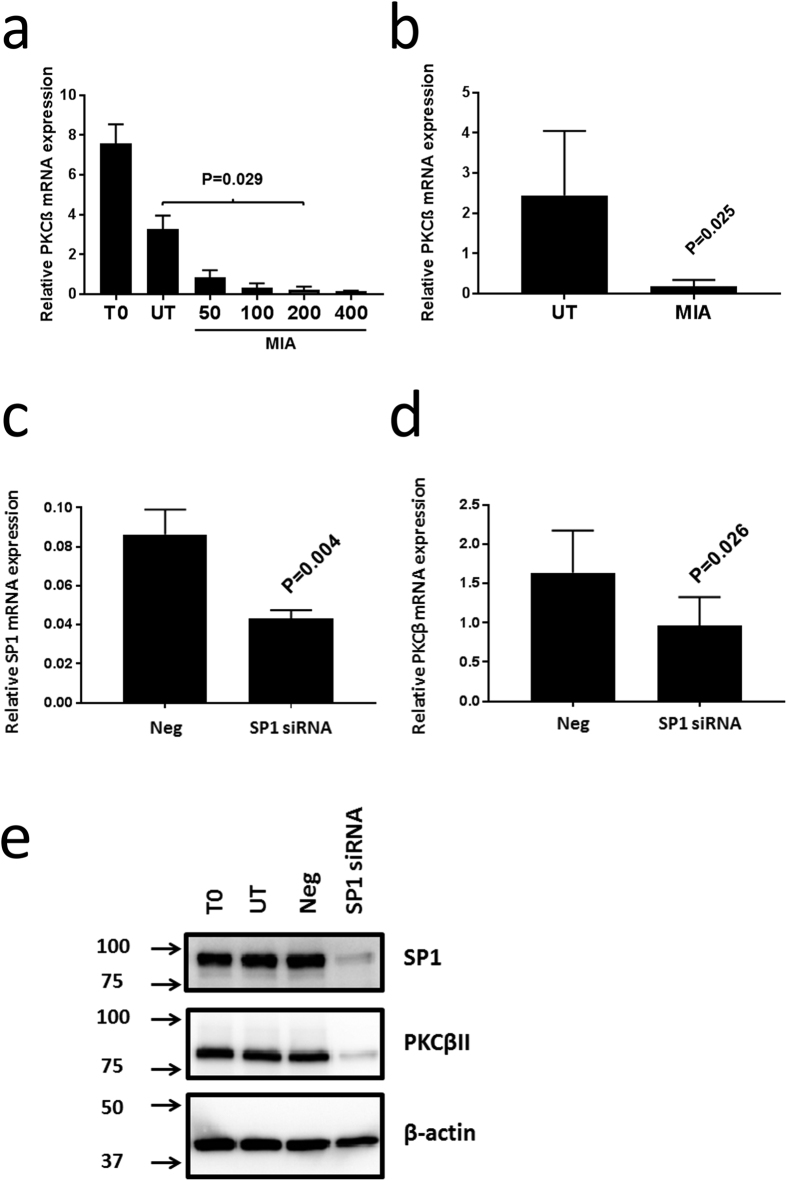
Mithramycin and SP1-specific siRNA reduce PKCβII mRNA and protein levels in CLL cells. 1 × 10^7^ CLL cells were cultured for 24 h in the presence of indicated concentrations of mithramycin (in nmol/L), or were nucleofected with the indicated SP1-specific or control siRNA oligonucleotides (Neg) and then cultured as described in the materials and methods. PKCβII mRNA levels were then measured by qRT-PCR and are reported relative to RNApolII expression. (**a**) PKCβII mRNA levels in CLL cells from a single patient. T0 indicates CLL cells used directly after thawing. UT indicates CLL cells cultured for 24 h. MIA indicates CLL cells cultured for 24 h with the indicated concentrations of mithramycin (in nmol/L). The results show mean ± SE of n = 3 separate experiments. (**b**) Effect of 200 nM mithramycin on PKCβII mRNA levels in CLL cells taken from 5 patients (mean ± SD). (**c**) Effect of SP1 siRNA compared to negative control siRNA (Neg) on primary CLL cells with respect to SP1 mRNA (mean ± SD of n = 4 experiments). (**d**) Effect of SP1 siRNA compared to negative control siRNA (Neg) on primary CLL cells with respect to PKCβ mRNA (mean ± SD of n = 3 experiments). (**e**) Western blot showing the effect of SP1 siRNA and negative control siRNA (Neg) on primary CLL cells with respect to SP1 and PKCβII protein levels (n = 1 experiment). Western blots (cropped images) were performed using 10 μg of total cellular protein derived from CLL cell lysates. In Parts **a** and **b** mithramycin treatment had no effect on overall CLL cell viability. In Parts **c** and **d**, CLL cell viability was equivalent between control and SP1-specific siRNA treated cells. Statistical analysis for this figure was performed using a student’s t-test for paired data.

Our previous work showed that treatment of CLL cells with mithramycin, a drug that intercalates into G-C rich areas of DNA to inhibit SP1-mediated gene transcription^29^,^30^, quantitatively reduces levels of PKCβII mRNA without affecting cell viability^24^. Our present work confirms these data, and shows that PKCβII mRNA levels in CLL cells are reduced in a concentration-dependent fashion by mithramycin (Fig. 1a). Likewise, mithramycin treatment of MEC1 cells, a B cell line derived from a CLL patient undergoing prolymphocytoid transformation^31^, showed similar concentration-dependent reduction in PKCβII mRNA regardless of whether the cells were cultured under serum-free or serum-rich conditions (Supplementary Figure 1A). Because SP1 transcribes many genes involved in cell cycle^32^, the use of serum-free conditions to culture MEC1 cells rules out any effects imparted by potential interruption of the cell cycle by mithramycin. We observed that maximal reduction of PKCβII mRNA levels in CLL and MEC1 cells was achieved using a concentration of 200 nM mithramycin (Fig. 1a and b, Supplementary Figure 1A). Taken together, these data show that MEC1 and CLL cells respond similarly to mithramycin, and suggest that the former cells can be used to model the behaviour of *in-vitro* cultured CLL cells.

To more directly examine the role of SP1 in the transcription of *PRKCB* we used siRNA. Figure 1c–e shows that reduction of SP1 mRNA and protein levels in CLL cells using specific siRNA results in a concomitant reduction of PKCβII mRNA and protein expression. Similar reduction of SP1 and PKCβII mRNA was observed using MEC1 cells, with optimal results being obtained using a mixture of SP1-specific siRNA oligonucleotides (Supplementary Figure 1B,C).

**Figure 2:**
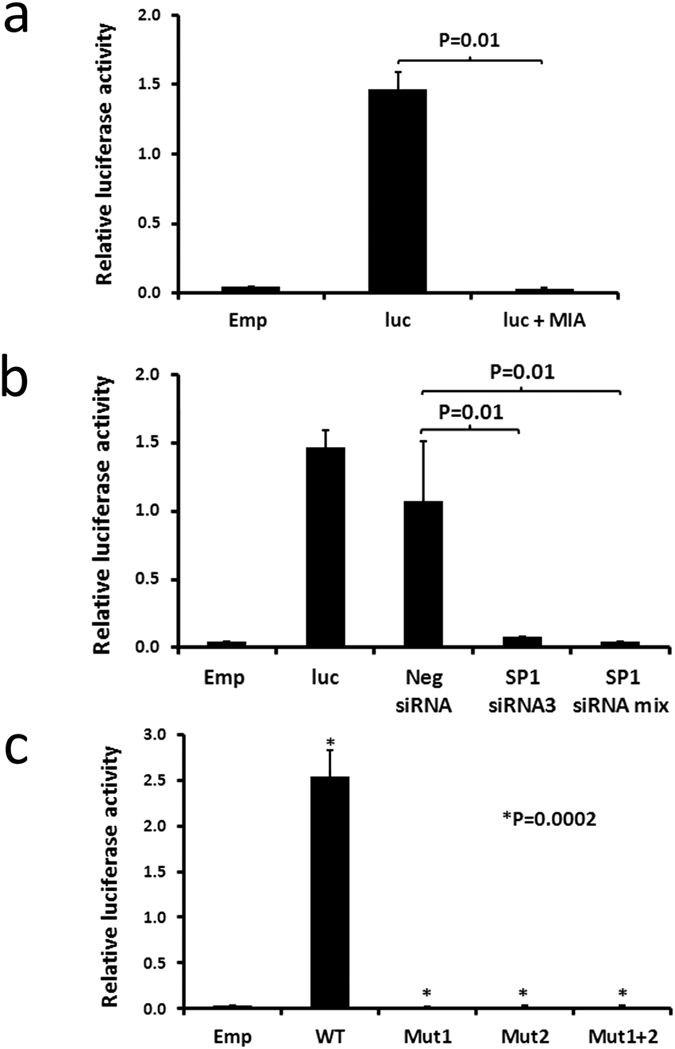
*PRKCB* promoter-driven luciferase expression in MEC1 cells is mediated by SP1. 2 × 10^6^ MEC1 cells were transfected either with empty pGL3 and pRL (Emp), or with pGL3-pkcβ-0.5 and pRL (luc) according to the procedure outlined in the materials and methods. (**a**) Effect of mithramycin. Cells were cultured for 24 h under serum-rich conditions, and then transferred into serum-free conditions for a further 48 h. For the final 24 h, 200 nM mithramycin (MIA) was added where indicated. (**b**) The effect of siRNA knockdown of SP1 expression was performed by co-transfection of the cells with either control or SP1-specific siRNA as indicated. Following culture for 72 h, the cells were harvested and a luciferase assay was performed. (**c**) The effects of site directed mutagenesis of the SP1 binding sites in the *PRKCB* promoter was investigated. MEC1 cells were transfected with pGL3 (Emp), wt pGL3-pkcβ-0.5 (WT), or with pGL3-pkcβ-0.5 containing a mutation within the SP1 binding site 1 (Mut 1), site 2 (Mut 2) or site 1 and 2 (Mut 1 + 2). Luciferase assays were performed following 72 h culture of the cells under serum-rich conditions. In all parts of this figure the data presented represent mean ± SE of n = 3 replicate experiments. Statistical analysis was performed using a students t-test for paired data.

We next investigated the role of SP1 in driving *PRKCB* promoter function by luciferase assay whereby 500 bp of the proximal *PRKCB* promoter responsible for its basal activity was cloned upstream of the luciferase gene in a pGL3 plasmid (pGL3-pkcβ-0.5 kb)^19^,^26^. Figure 2a shows that presence of 200 nM mithramycin significantly reduces the level of luciferase activity in transfected MEC1 cells that were cultured under serum-free conditions. Reduction of SP1 expression with specific siRNA also blocked *PRKCB* promoter-driven expression of luciferase in MEC1 cells, whereas control siRNA or mock transfection had no effect (Fig. 2b). The *PRKCB* promoter contains two binding motifs for SP1 at positions −94 (site 1) and −63 (site2)^19^. Loss of either or both of these motifs through site directed mutagenesis resulted in a complete loss of promoter function (Fig. 2c). This indicates that both SP1 motifs are essential for *PRKCB* basal promoter activity and supports previous reports demonstrating that synergistic binding of SP1 drives transcriptional activation in gene promoters^33^,^34^. Taken together, these results demonstrate a direct relationship between SP1 and *PRKCB* gene transcription, and shows that synergistic binding of SP1 is essential for PKCβII expression in CLL cells.

### SP1 binds more readily to the PRKCB promoter sequence in CLL than normal B cells

**Figure 3:**
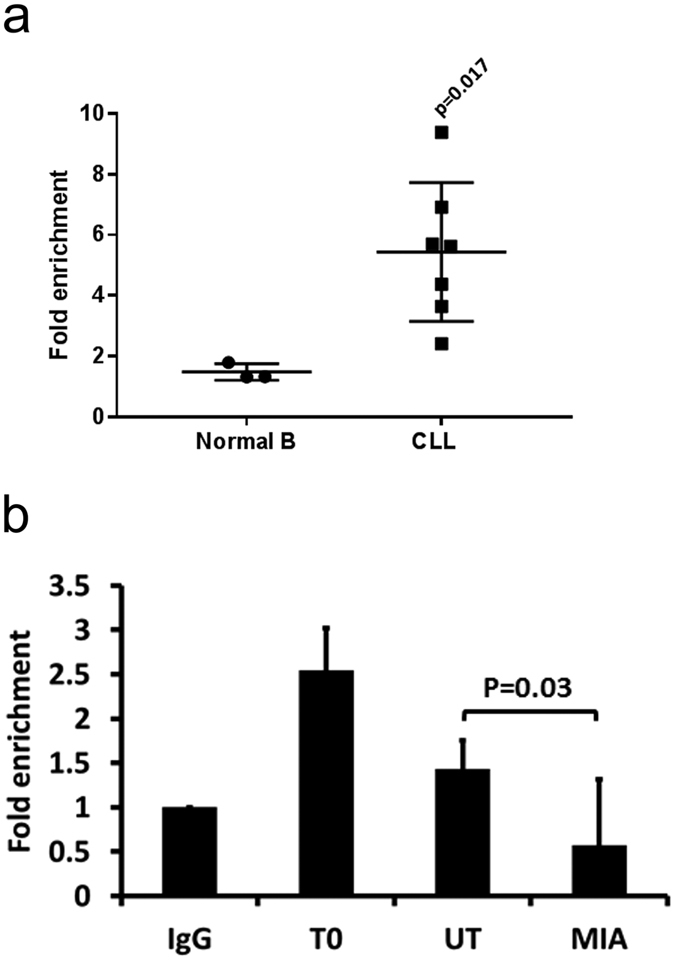
SP1 binds to the *PRKCB* promoter sequence in CLL cells. ChIP analysis of SP1 binding to the *PRKCB* promoter. (**a**) CLL and normal B cell extracts from 5 × 10^6^ cells were prepared and SP1 was immunoprecipitated. *PRKCB* promoter sequences associated with SP1 were detected by qPCR and are presented as fold enrichment compared to the *PRKCB* promoter sequences associated with the non-specific IgG immunoprecipitation control. The mean ± SD of these experiments is displayed. Statistical analysis was performed using a Mann-Whitney U-test. (**b**) 5 × 10^6^ CLL cells were used either immediately after thawing (T0), or were incubated for 24 h in the absence (UT) or presence of 200 nM mithramycin (MIA). SP1 was immunoprecipitated from prepared extracts and the presence of the *PRKCB* promoter was detected using qPCR. The results are presented as fold enrichment of *PRKCB* promoter sequences associated with SP1 compared to the IgG immunoprecipitation control (IgG). The data presented represent mean ± SE of n = 3 replicate experiments using cells from the same patient. Statistical anlaysis was performed using a students t-test for paired data.

A direct role of SP1 in regulating PKCβII expression was confirmed using chromatin immunoprecipitation (ChIP). We found that SP1 bound the *PRKCB* promoter in CLL cells, and that this binding was significantly enhanced compared to that in normal B cells (Fig. 3a). In line with the ability of mithramycin to suppress PKCβII expression in CLL cells, we also found that SP1 binding to the *PRKCB* promoter is disrupted in cells treated with this compound (Fig. 3b). Thus, SP1 binds the *PRKCB* promoter in CLL cells and its increased association is likely responsible for overexpression of the gene.

### The PRKCB promoter sequence in CLL cells is unmethylated and contains higher levels of histone marks permissive of gene activation

The *PRKCB* promoter is enriched with CpG islands and expression from this gene can potentially be affected by gene methylation^19^,^22^. To investigate whether this contributes to enhanced binding of SP1 to *PRKCB* in CLL cells we performed experiments assessing the methylation status of the CpG island located near the SP1 binding sites of the promoter. We found that this region was virtually unmethylated and markedly similar between CLL and normal B cells (p = 0.84, Mann-Whitney U-test, Table 1).

**Figure 4:**
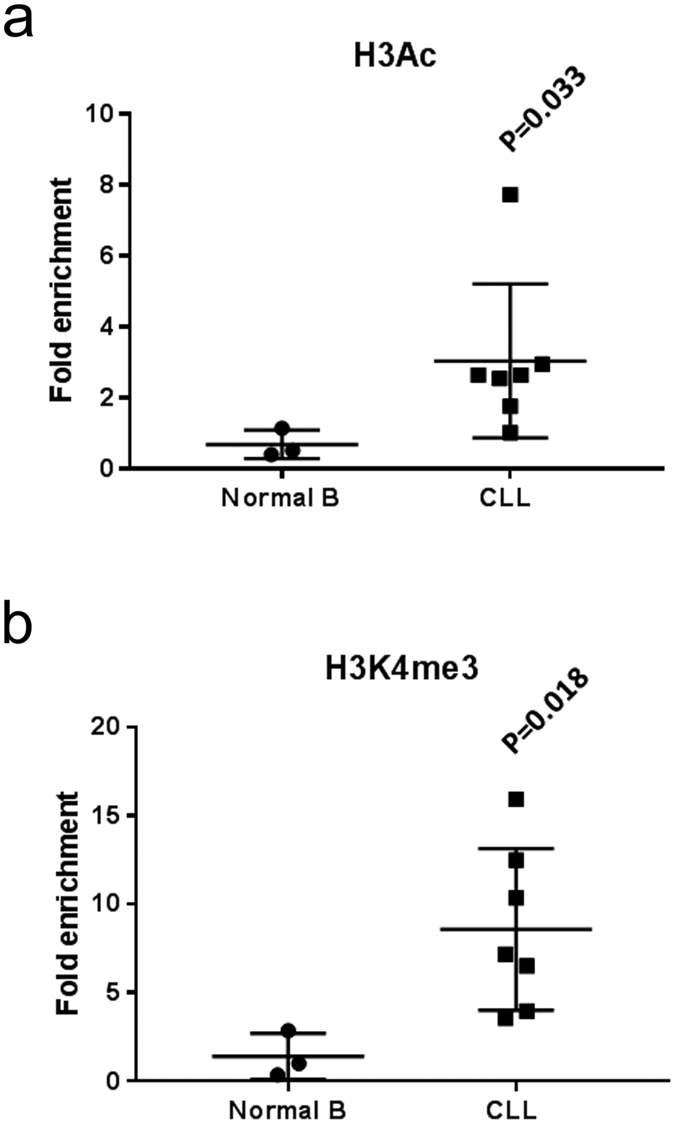
The *PRKCB* promoter of CLL cells contains histone marks permissive of gene activation. Purified normal B cells and CLL cells were analysed by ChIP for H3Ac and H3K4me3 histone mark association with the promoter region of *PRKCB* upstream of the transcriptional start site. (**a**) Comparison of H3Ac histone mark association with *PRKCB* promoter in normal B and CLL cells. (**b**) Comparison of H3K4me3 histone mark association with *PRKCB* promoter in normal B and CLL cells. The results are presented as fold enrichment of *PRKCB* promoter sequences associated with H3Ac or H3K4me3 compared to the IgG immunoprecipitation control, and represent the mean ± SD using cells from different patients. Statistical analysis was performed using a Mann-Whitney U-test.

Histone H3 hyperacetylation (H3Ac) and trimethylation of K4 (H3K4me3) are both chromatin marks that facilitate the formation of an “open” structure of chromatin that is conducive to increased access of TFs and induction of transcription^35^,^36^. We used ChIP to investigate the extent of H3Ac and H3K4me3 marking associated with the *PRKCB* promoter in CLL and normal B cells, focussing on the region of DNA containing the SP1 binding sites (Fig. 4a and b). We found that these histone marks were present to a significantly greater extent with the *PRKCB* promoter in CLL cells compared to normal B cells. This suggests that increased access of SP1 to the *PRKCB* promoter in CLL cells compared to normal B cells is likely the result of histone relaxation due to permissive epigenetic marking rather than changes in gene methylation.

### VEGF induces PKCβ gene expression in CLL cells by stimulating SP1 and inhibiting STAT3 association with the *PRKCB* promoter sequence

**Figure 5:**
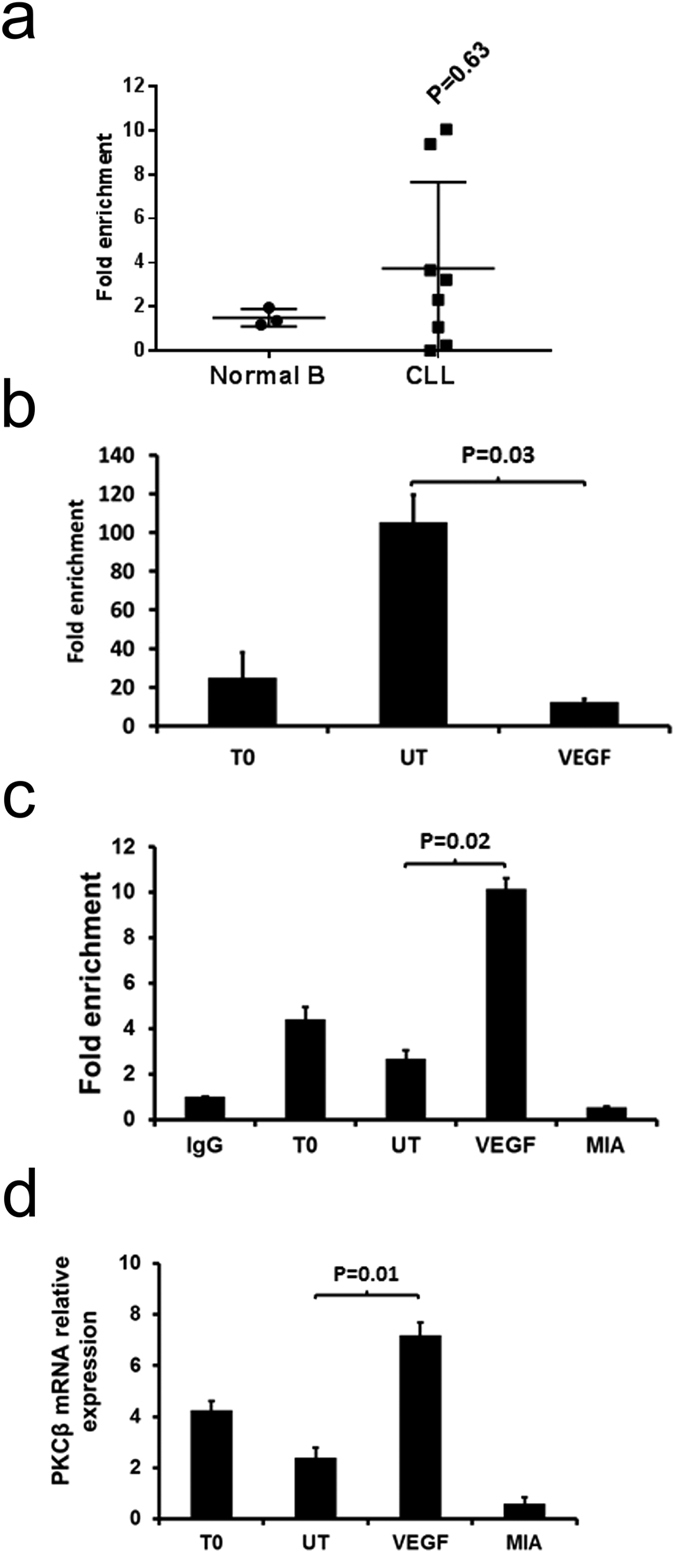
VEGF stimulates SP1 association with the *PRKCB* promoter sequence in CLL cells. 5 × 10^6^ CLL cells were used directly (T0) or cultured overnight in the absence (UT) or presence of 100 ng/mL VEGF or 200 nM mithramycin (MIA). (**a**) ChIP analysis of STAT3 association with the *PRKCB* promoter in CLL and normal B cells in individual samples. The mean ± SD of these experiments is displayed. Statistical analysis was performed using a Mann-Whitney U-test. (**b**) ChIP analysis of STAT3 association with the *PRKCB* promoter in CLL cells incubated overnight ± VEGF. (**c**) ChIP analysis of SP1 association with the *PRKCB* promoter using the same CLL samples as in part (**b**). IgG is the immunoprecipitation control. (**d**) qRT-PCR analysis of PKCβII mRNA levels in CLL cells measured in comparison to RNApolII. For ChIP analyses *PRKCB* promoter sequences associated with STAT3/SP1 were detected by qPCR and are presented as fold enrichment compared to the *PRKCB* promoter sequences associated with the non-specific IgG immunoprecipitation control. In parts (**b,c** and **d**) the data presented represent the mean ± SE of n = 3 experiments using CLL cells from different patients. Statistical analysis was performed using a students t-test for paired data.

Derepression of gene expression may also explain the high levels of PKCβII in CLL cells, and in this respect a recent study demonstrated that active STAT3 can interact with the *PRKCB* promoter to suppress expression of this gene in myeloid cells^23^. We used ChIP to compare STAT3 binding to the *PRKCB* promoter in CLL and normal B cells and we found no significant difference in overall association (Fig. 5a). However, there did seem to be greater variability in STAT3 binding between CLL cases, with some having quite high levels while others had very little. Interestingly, overnight culture of CLL cells resulted in a spontaneous increase in STAT3 association with the *PRKCB* promoter (Fig. 5b), and this corresponded with both an observed decrease in *PRKCB* promoter-associated SP1 (Figs 3b and 5c) and with the dynamics of PKCβII mRNA levels in CLL cells cultured overnight (Figs 1b and 5d). To investigate this phenomenon we referred to previous work demonstrating VEGF-induction of PKCβII expression in CLL cells^24^. Figure 5d confirms within the current study that addition of VEGF to cultures of CLL cells stimulates PKCβII mRNA production, and Fig. 5c and b further demonstrate by ChIP analysis that the mechanism respectively involves enhancement of SP1 and repression of STAT3 binding to the *PRKCB* promoter. We confirmed that STAT3 operates as a repressor of *PRKCB* function in B lymphoid cells in experiments using a pGL3-pkcβ1.2 kb construct containing reported STAT3 binding sites^23^. Similar to the findings of this group, we found that mutation of STAT3 binding site 1 or sites 2 plus 3 had no effect, whereas mutation of STAT3 binding site 4 resulted in significantly increased promoter activity (Supplementary Figure 2). Taken together, these results strongly suggest a dynamic relationship between gene expression mediated by SP1 and gene repression mediated by STAT3 in the control of *PRKCB* transcription in CLL cells that is regulated by the presence of VEGF.

### Higher levels of SP1 expression in CLL cells likely contributes to overexpression of PKCβII

**Figure 6:**
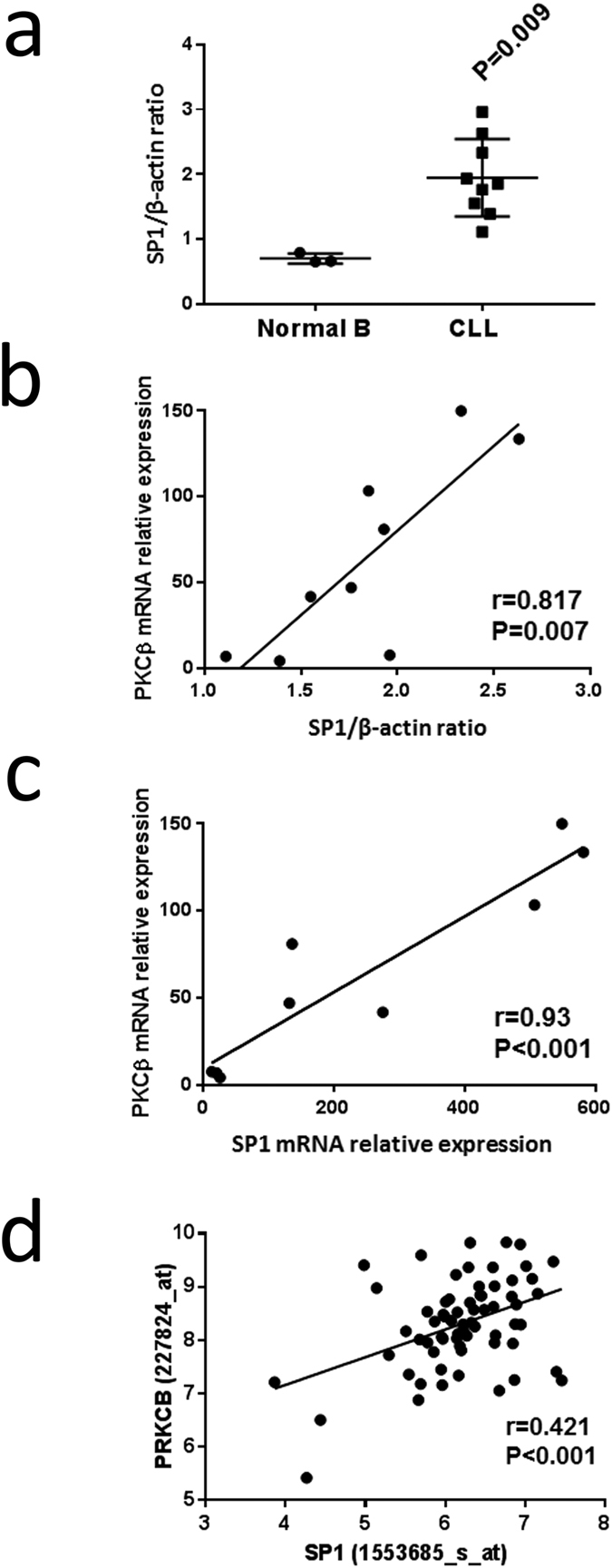
SP1 is overexpressed in CLL cells, and expression correlates with *PRKCB* transcription. (**a**) SP1 protein expression relative to β-actin was determined in lysates of purified normal B and CLL cells by Western blot analysis. 10 μg of protein was used for each sample, and the ratio of SP1 to β-actin was determined following imaging of chemoluminescence. Statistical analysis was performed using a Mann-Whitney U-test. (**b**) Graph showing the relationship between SP1 protein expression and PKCβ mRNA levels, determined by qRT-PCR, in purified CLL cells. (**c**) Graph showing the relationship between SP1 and PKCβ mRNA levels, determined by qRT-PCR, in purified CLL cells. (**d**) Graph showing relationship between *SP1* and *PRKCB* gene expression in CLL cells taken from publically-available data stored on the Immuno-Navigator database^37^.

We next considered whether high levels of SP1 contributed to overexpressed PKCβII in CLL cells. We found that SP1 expression was significantly higher in CLL cells than in normal B cells (Fig. 6a), and that SP1 protein and mRNA levels correlated highly with PKCβII mRNA expression within our local cohort of patient samples (Fig. 6b and c). Furthermore, reanalysis of gene expression profile data available for CLL cells within the Immuno-Navigator database^37^ also showed correlation between *SP1* and *PRKCB* gene expression (Fig. 6d). Taken together, these data strongly suggest a direct relationship between SP1 and *PRKCB* gene expression in CLL cells.

## Discussion

In this manuscript we describe the mechanism of VEGF-stimulated PKCβ gene expression on the transcriptional level. The results we present are the first to demonstrate a clear role for SP1 in the regulation of PKCβII expression in CLL cells, and we suggest that access of SP1 to the promoter of *PRKCB* likely results both from a chromosome landscape that is permissive of gene transcription and from VEGF-mediated inhibition of the suppressive effects of STAT3. These findings are not only important to our understanding of the pathobiology of CLL cells, but may also be relevant for the microenvironmental stromal cells where CLL cells are reported to induce expression of PKCβII^38^ and for the malignant cells of other neoplasms where PKCβII is overexpressed^12^,^13^,^14^,^15^.

SP1 is a ubiquitous TF that binds GC-rich regions of target gene promoters and transcribes a variety of genes involved in cell cycle progression, differentiation, growth and apoptosis^32^. We have found that SP1 leads to transcription of *PRKCB*, a finding that brings insight to previous studies characterising the basal promoter region of this gene^18^,^19^. Our findings confirm those of Hagiwara *et al*.^22^ who also use mithramycin and SP1-specific siRNA to investigate PKCβ expression in HeLa cells. However, our data provide further understanding by showing that SP1 directly binds and induces transcription from the PKCβ gene promoter within primary CLL cells. Furthermore, our study also differs from the study by Hagiwara *et al*.^22^ because we find largely similar DNA methylation of the *PRKCB* promoter region containing SP1 binding sites in normal B compared to CLL cells, and this finding is supported by those of another study comparing the DNA methylome between normal B and CLL cells^39^. Instead, greater access of SP1 to the *PRKCB* promoter in CLL cells seems to be due to a different epigenetic mechanism. H3Ac and H3K4me3 are both euchromatin histone marks permissive of active gene expression^35^,^36^, and we show that these marks are associated to a greater extent with the *PRKCB* promoter in CLL cells than in normal B cells. Histone marks have not previously been investigated within the context of PKCβ gene regulation in CLL cells or other cell types, but are nevertheless important because of their role in determining chromatin structure and state of cellular differentiation. Recent reports suggest that expression of enzymes responsible for histone marking is deregulated in CLL, and may relate to disease prognosis and pathogenesis^40^,^41^,^42^. One consequence of this deregulated expression may be the granting of greater access of SP1 to the promoter region of *PRKCB*.

Overexpression of PKCβII in CLL cells is, at least, partially the result of concomitant increased expression of SP1. This conclusion is supported by observations by us and others^43^ that this TF is expressed at significantly higher levels in CLL cells than in normal B cells, and by our ability to correlate SP1 protein and mRNA levels with PKCβ mRNA levels in CLL cells. In particular, the relationship between SP1 and PKCβ mRNA expression is strongly supported by our analysis of publicly-available gene expression profiles associated with CLL cells available through the Immuno-Navigator website^37^. Although correlation of *SP1* with *PRKCB* expression does not mean that one affects the other, our demonstration that SP1 association with the *PRKCB* promoter correlates with decreased and increased gene expression strongly suggests that SP1 overexpression plays a distinct role in increased PKCβ expression in CLL cells. This finding therefore provides insight into observations of increased SP1 expression in other tumours such as those of the lung where it contributes to tumour progression, particularly in early stages of the disease^44^, and where this TF likely also drives the increased levels of PKCβ expression that have been reported in these tumours^45^,^46^.

An additional factor contributing to overexpression of PKCβII is suggested by our previous observation that VEGF stimulates *PRKCB* transcription in CLL cells^24^. The experiments presented within the current manuscript bring insight into the mechanism of how this happens, and provides a potential explanation for reported observations of overexpressed PKCβII in tissue sections from B lymphoid malignancies^13^,^47^. We show that PKCβII mRNA levels and SP1 association with the *PRKCB* promoter decrease with overnight culture of CLL cells, and that this is coupled with an increased association of STAT3. When VEGF is present in CLL-cell cultures, SP1 association with the *PRKCB* promoter is maintained/stimulated whilst STAT3 association remains low or is suppressed. This mechanism is likely to involve active PKCβII because of our previous work demonstrating that pretreatment of CLL cells with LY379196, a PKCβ-specific inhibitor^48^, blocks the effects of VEGF^24^. That STAT3 is able to interact with the *PRKCB* promoter in CLL cells is supported by studies showing constitutive activation of this protein^49^,^50^. Taken together, these observations potentially explain how active PKCβII regulates its own gene expression^25^,^26^, and also link SP1 and STAT3 into the pathobiology of CLL because high levels of VEGF are present in lymph nodes and bone marrow of CLL patients with late stage disease^28^.

Other TFs reported to regulate *PRKCB* transcription include MITF and RUNX1^20^,^21^, but these TFs are likely less important than SP1 in regulating PKCβ expression in CLL cells. Direct binding of MITF to the *PRKCB* promoter has not been demonstrated, and it is proposed that this TF acts as a co-activator^20^. With respect to RUNX1, ChIP analysis has demonstrated binding of this TF to the *PRKCB* promoter but mutation of the RUNX1 binding site within this promoter only partially inhibits transcription of the gene^21^. The experiments using site-directed mutagenesis of the SP1 binding sites to completely block gene expression driven by this promoter suggests that basal transcription of *PRKCB* requires SP1 but not RUNX1. It is possible that RUNX1 may cooperate with SP1 to promote PKCβ gene expression in CLL cells because SP1 is shown to interact with the DNA binding domain of RUNX1 to facilitate expression of other genes in other cell types^51^. Whether this is the case in CLL and *PRKCB* will require further experiments using larger promoter constructs.

A potential limitation of our study is the use of fold enrichment to normalise the data obtained from the ChIP experiments. This technique of normalisation is open to variation between primer sets, samples and experiments as described by Haring *et al*.^52^. However, we believe our experimental approach limited these potential influences. Our experiments using mithramycin are robust because we observed that this agent effectively displaced SP1 from its binding sites within the proximal promoter of *PRKCB* both in MEC1 and in primary CLL cells, an observation consistent with reported effects of this drug on this^22^ and other genes^29^,^30^. Moreover, potential inter-experimental variance was reduced by performing each ChIP experiment on all CLL cell samples in one go. Although each patient sample was slightly different, variation in SP1/STAT3 binding was observed to be consistent with variation in PKCβ mRNA levels, particularly with respect to cells incubated ±VEGF.

In conclusion, this is the first paper to demonstrate a clear role for SP1 in *PRKCB* transcription, and further link this TF into the pathobiology of CLL cells. Because of the myriad of genes that are potentially transcribed by SP1^32^, future work identifying the mechanism(s) controlling SP1 interaction with the promoter region of *PRKCB*, particularly with respect to how STAT3 may regulate this interaction, will not only be important for understanding how this gene is regulated in the malignant cells of CLL and other diseases where PKCβ is overexpressed, but may also add important insight into the regulation of other genes important to the pathobiology of these diseases.

## Methods

### Cell culture

CLL cells were obtained from the peripheral blood taken from patients with informed consent and with the approval of the Liverpool Research Ethics Committee. Stored CLL cell samples from the Liverpool Leukaemia Biobank were prepared using a standard protocol as described previously^53^, and had a minimum viability of 80%. CLL sample usage and performed experiments on these samples were recorded in compliance with a Research Ethics Agreement overseen by the University of Liverpool and Royal Liverpool and Broadgreen NHS University Hospital Trust.

MEC-1 cells were obtained from the Leibniz Institute DSMZ-German Collection of Microorganisms and Cell Cultures (Braunschweig, Germany) and cultured in Dulbecco’s Modified Eagle Medium (DMEM). Mithramycin was purchased from Sigma-Aldrich (Gillingham, U.K.).

### Quantitative reverse transcriptase PCR

ZR-RNA™ MiniPrep kits (Zymo Research, Cambridge Bioscience Ltd, Cambridge U.K.) were used to isolate total RNA, which was then quantified using a Nanodrop 2000C spectrophotometer (NanoDrop Products Wilmington, DE, USA). cDNA was generated using 1 μg of RNA with an oligo-dT primer and Moloney murine leukemia virus reverse transcriptase (Promega, Southampton, UK). PCR primers were purchased from Eurofins (MWG Operon, Ebersberg, Germany) and are listed in Table 2. Reactions were carried out using Hot Fire pol EvaGreen qPCR mastermix (Newmarket Scientific Ltd, U.K.) on a Stratagene MX3000P PCR machine with the following cycle conditions: 10 min at 95 °C then 40 cycles of 30 sec at 95 °C, 20 s at 58 °C (PKCβII) or 64 °C (SP1), 30 s at 72 °C and 11 s at 81 °C (PKCβII) or 80 °C (SP1) to collect fluorescence data. Melt curve analysis was performed to assess purity of the amplified products. Reactions were normalised to RP2 and relative expression determined using the ∆∆Ct method.

### DNA methylation analysis

Purified normal B and CLL cells were isolated using anti-CD19 magnetic MicroBeads and the MiniMacs system according to the manufacturer’s instructions (Miltenyi Biotech Ltd, Bisley, UK). Cell purity was assessed by flow cytometry using CD20-PE antibodies (BD Biosciences, UK), and samples with populations >90% CD20^+^ cells were considered to be of sufficient purity for subsequent experiments. Genomic DNA from purified cells was extracted using a Promega Wizard genomic DNA extraction kit (Promega, Madison, WI, USA). 1 μg of genomic DNA was sodium bisulphite treated using a EZ-DNA methylation gold kit (Zymo Research, Irvine, CA, USA). A CpG-rich target region within the *PRKCB* promoter was selected for interrogation, and forward, reverse and pyrosequencing primers designed using Pyromark Assay Design 2.0 software (Qiagen, Valencia, CA, USA) and synthesized by Eurofins MWG Operon (Table 2). PCR amplification was performed using 400 μM forward and reverse primers, 60 ng bisulphite-treated DNA, 200 μM dNTPs, 1 mM MgCl_2_ and 1.25 U GoTaq Flexi DNA polymerase (Promega, Madison, WI, USA). PCR cycling conditions were as follows: 94 °C for 3 min, followed by 40 cycles of 94 °C for 30 s, 50 °C for 30 s and 72 °C for 30 s and an additional 72 °C extension for 10 min. Specific PCR product quality and quantity were confirmed by agarose gel electrophoresis. PCR products were then immobilized on to streptavidin sepharose beads (GE Healthcare Biosciences, Pittsburgh, PA, USA) and sequentially washed in 70% ethanol, 0.2 M NaOH and 10 mM Tris acetate, pH 7.5, using a PSQ96 Vacuum Workstation (Qiagen, Valencia, CA, USA). PRCKBmeth_seq primer was then hybridized to the retained biotinylated DNA strand in annealing buffer and analyzed using PSQ96 MA Pyrosequencer and PyroMark Gold Q96 reagents (Qiagen, Valencia, CA, USA). Results presented as % methylation represent the mean average methylation of 8 CpGs within the analyzed sequence.

### Nucleofection and luciferase assays

All cell transfections were performed using an Amaxa nucleofector (Lonza Biologics plc, Tewkesbury UK). MEC-1 cells were transfected using solution V and programme X-01. A plasmid (pGL3-pkcβ-0.5) containing 500 bp of the proximal promoter of *PRKCB* cloned upstream of the firefly luciferase was kindly provided by Dr A P Fields (Mayo Clinic College of Medicine, Jacksonville, Florida, USA)^26^. Plasmids wt-pGL3-pkcβ-1.2 kb and site 1, site 2 + 3, or site 4 mutants of the STAT3 binding sites within the *PRKCB* promoter of pGL3-pkcβ-1.2 kb were prepared as described^23^. MEC-1 (2 × 10^6^ cells/ml) were co- transfected with 2 μg of pGL3-pkcβ-0.5 and 100ng of pRL Renilla luciferase control plasmid (Promega, UK) to allow for normalisation of transfection efficiency. In some experiments SP1-specific siRNA duplexes were also included (see below). Firefly and Renilla luciferase activity was measured using a Dual-Luciferase Reporter Assay kit (Promega, UK), and luciferase activity of the former is reported relative to that the latter.

### siRNA knockdown of SP1

siRNA (TriFECTa^®^ RNAi, Integrated DNA Technologies, Glasgow U.K.) was used to knockdown expression of SP1 in CLL, MEC-1 and Daudi cells at a final concentration of 2 μM. The sequences for the SP1-specific siRNA oligonucleotide duplexes are displayed in Table 2. Twenty four hours after transfection, cells were washed once with PBS and incubated for 24 hours in serum rich media, which was then replaced with serum free media and incubation for a further 48 hours. Knockdown of SP1 expression was confirmed by Western blot and qRT-PCR analysis.

### Site-directed mutagenesis

Site-directed mutagenesis of the SP1 binding sites within the *PRKCB* promoter sequence was carried out using a Stratagene QuickChange Site-Directed Mutagenesis kit (Agilent Technologies, Stockport, UK) according to manufacturer’s instructions. The primers used for introducing the mutations were all from Eurofins MWG Operon (Table 2). Introduced mutations were verified by commercial sequencing (Source BioScience plc, Nottingham, UK).

### Chromatin immunoprecipitation (ChIP) analysis

Cells (5 × 10^6^) were washed with PBS and then treated with 0.5% formaldehyde for 5 min at room temperature to cross link proteins and DNA. The reaction was stopped with 135 mM glycine for 5 min at room temperature. Following washing with cold PBS, cells were lysed with 1 ml cold lysis buffer (10 mM Tris-HCl pH 7.4, 10 mM NaCl, 5 mM MgCl_2_, 0.2% NP-40 plus protease and phosphatase inhibitors) on ice for 15 min. Nuclei were isolated by centrifugation at 500 g for 5 min (4 °C), resuspended in 500 μl of cold MNase buffer (0.3 M sucrose, 10 mM Tris-HCl pH 7.4, 15 mM NaCl, 60 mM KCl, 5 mM MgCl_2_, 3 mM CaCl_2_, 0.5 mM spermidine, 0.15 mM spermine) to which 125 U micrococcal nuclease was added and then incubated for 20 min at 37 °C. The reaction stopped by the addition of EDTA to a final concentration of 10 mM. NP-40, sodium deoxycholate and SDS were added to final concentrations of 1%, 0.5% and 0.1% SDS respectively. Nuclei were disrupted by sonication to release fragmented chromatin and insoluble material removed by centrifugation at 12000 × g for 5 min (4 °C). Protein/DNA complexes were precipitated overnight at 4 °C using 5 μg ChIP-grade antibody and 20 μl Magna ChIP protein A + G magnetic beads (Millipore, Watford, UK). Following extensive washing, beads were resuspended in elution buffer (1% SDS, 0.1 M NaHCO_3_, 0.2 M NaCl, 10 μg proteinase K) and incubated at 65 °C for 2 h to reverse cross links. Eluted DNA was purified using a DNA clean and concentrator-5 kit (Cambridge Bioscience Ltd, UK). One microlitre of the purified DNA was used for PCR amplification using *PRKCB* promoter specific primers (Table 2). Cycle conditions were 20 s at 95 °C then 40 cycles of 20 s at 56 °C, 30 s at 72 °C and 11 s at 85 °C to collect fluorescence data.

### Gene expression profile (GEP) analysis

Publicly available GEP data for *SP1* and *PRKCB* expression in CLL cells was analysed using the Immuno-Navigator website (http://sysimm.ifrec.osakau.%0dac.jp/immuno-navigator)^37^. Jetset (http://www.cbs.dtu.dk/biotools/jetset/)^54^ was employed to select reliable probes for *SP1* and *PRKCB* from the Affymetrix hgu133plus2 chip used to construct the GEP data. Pearson’s product-moment correlation analysis was performed using GraphPad Prism™ software.

## Additional Information

**How to cite this article**: Al-Sanabra, O. *et al*. Transcriptional mechanism of vascular endothelial growth factor-induced expression of protein kinase CβII in chronic lymphocytic leukaemia cells. *Sci. Rep.* **7**, 43228; doi: 10.1038/srep43228 (2017).

**Publisher's note:** Springer Nature remains neutral with regard to jurisdictional claims in published maps and institutional affiliations.

## Supplementary Material

Supplementary Data

## Figures and Tables

**Table 1:** PRKCB promoter methylation in normal B and CLL cells.

Sample ID	B-CLL/Normal B	% purity	Mean % methylation
***2649***	Normal B	96.12	**3.49**
***2667***		97.12	**2.22**
***2668***		93.15	**2.3**
***2675***		—	**1.73**
***2063***	B-CLL	99.79	**3.32**
***2064***		—	**4.51**
***2226***		—	**2.31**
***2262***		93.03	**1.05**
***2458***		91.09	**0.8**
***2536***		99.33	**3.16**

Normal B and CLL cells were purified each from 5 healthy donors or CLL patients, respectively. Genomic DNA was isolated from these cells, and methylation of the *PRKCB* promoter was determined as described in the materials and methods. % methylation is reported as the mean average methylation of 8 CpGs within the analyzed sequence. Where a dash occurs within the % purity of the cells indicates where cell purity was not assessed following purification.

**Table 2:** Sequences of DNA oligonucleotides used.

**qRT PCR Primers**		
**PKCβII**	Forward	5′-TGGGGTGACACCCAAGACATTC-3'
	Reverse	5′-GCTGGATCTCTTTGCGTTCAAG-3'
**SP1**	Forward	5′-GACGTTGATGCCACTGTTGGCAAG-3'
	Reverse	5′-TCAAGACCCACCAGAATAAGAAGGGAG-3'
**RNA polymerase II (RP2)**	Forward	5′-CAAGACTGCTGAGACTGGATAC-3'
	Reverse	5′-CAAAGCGGAACTTCTTCTCAAAAG-3'
**DNA methylation Primers**		
**PRKCBmeth**	Forward	5′-GTTTGGGTATATTTTTTGAA-3'
	Reverse	5′-Biotin-CCCTCCTCATTTACATC-3'
	Sequence	5′-TTGGGTATATTTTTTGAA-3'
**Site-Directed Mutagenesis Primers**		
**SP1 m1**	Forward	5′-AGCAGCTGGCAGCGCTATGCTAGGCCTGGGCGCG-3'
	Reverse	5′-CGCGCCCAGGCCTAGCATAGCGCTGCCAGCTGCT-3'
**SP1 m2**	Forward	5′-TGGGCGCGATGCAAATGAGGAATGCTAGGCTGGCCCGGG-3'
	Reverse	5′-CCCGGGCCAGCCTAGCATTCCTAGCATTCCTCATTTGCATCGCGCCCA-3'
**ChIP Primers**		
**PRKCB prom**	Forward	5′-GCACGCTTAGCCGCGAGG-3'
	Reverse	5′-AGCTGCTGCCGCTCGTCC-3'
**siRNA oligos**		
**Duplex 1**	Sense	5′-CCAAGGAAAUAAGGACAGUCUAGCT-3'
	Antisense	3′- AUGGUUCCUUUAUUCCU-GUCAGAUCGA-5'
**Duplex 2**	Sense	5′-CCCUCAACCCUAUUCAUUAGCAUTA-3'
	Antisense	3′-AUGGGAGUUGGGAUAAGUAAUCGUAAU-5'
**Duplex 3**	Sense	5′-GUGCAAACCAACAG-AUUAUCACAA-3'
	Antisense	3′- GUCCACGUUUGGUUGUCUAAUAGUGUU-5'
