# Supplementary material for: Transcriptional mechanism of vascular endothelial growth factor-induced expression of protein kinase CβII in chronic lymphocytic leukaemia cells
Source: Sci Rep. 2017 Feb 24;7:43228. doi: 10.1038/srep43228 (PMC5324130; doi:10.1038/srep43228)
Supplement: Supplementary Data [file srep43228-s1.pdf]

Supplementary Figures

Transcriptional mechanism of vascular endothelial growth factor-induced expression of protein kinase CβII in chronic lymphocytic leukaemia cells.

Ola Al-Sanabra, Andrew D. Duckworth, Mark A. Glenn, Benjamin R.B. Brown, Piera Angelillo, Kelvin Lee, John Herbert, Francesco Falciani, Nagesh Kalakonda, and Joseph R. Slupsky.

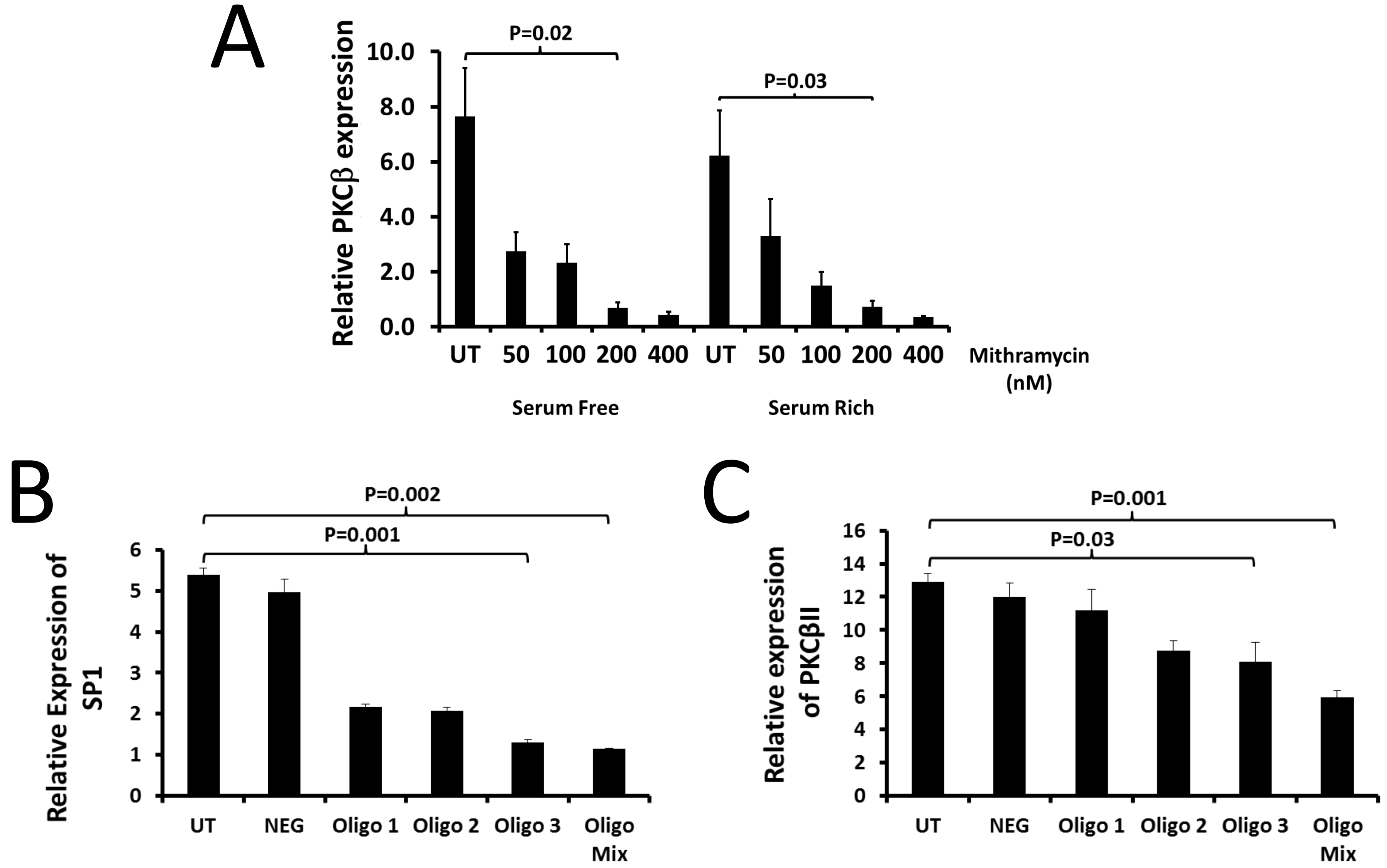

Supplementary Figure 1 *Treatment of Mec1 cells with mithramycin or SP1-specific siRNA reduces PKCß mRNA levels*. 2x10^{6} MEC1 were either were cultured for 24h in the presence of the indicated concentrations of mithramycin (in nmol/L), or were nucleofected with the indicated SP1-specific (Oligo 1, 2, 3 and mix) or control siRNA (NEG) oligonucleotides and then cultured as described in the materials and methods for each cell type. Harvested cells were were analyzed by qRT-PCR for SP1 (**A**, **B**) or for PKCβII mRNA (**C**) using RNApolII as a reference gene. The results presented represent the mean±SE of n=3 separate replicates. Statistical analysis for all parts in this figure were performed using a students t-test for paired data. UT - untreated

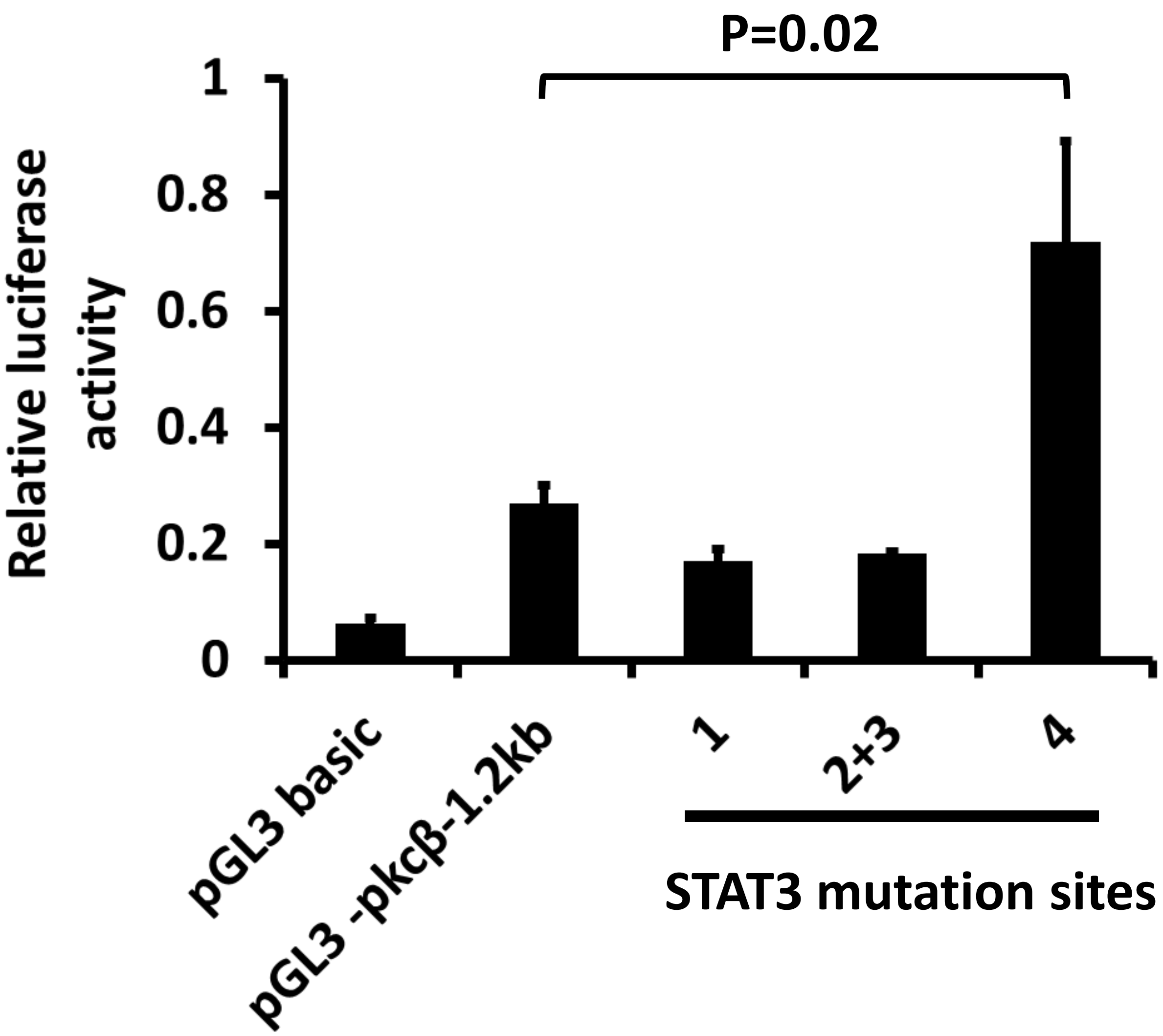

Supplementary Figure 2 *Analysis of STAT3 repression of PRKCB promoter function in Mec1 cells*. 2x10^6 MEC1 were cotransfected with (0.1µg) pRL and (2µg each) of wt pGL3-pkcβ-1.2kb, or site 1, site 2+3, or site 4 mutants of STAT3 binding sites within the *PRKCB* promoter of the pGL3-pkcβ-1.2kb construct. Luciferase assays were performed following 72h culture of the cells under serum-rich conditions, and are reported relative to renilla expression. The data represented mean±SE of three independent experiments. Statistical analysis was performed using a student's t-test for paired data.

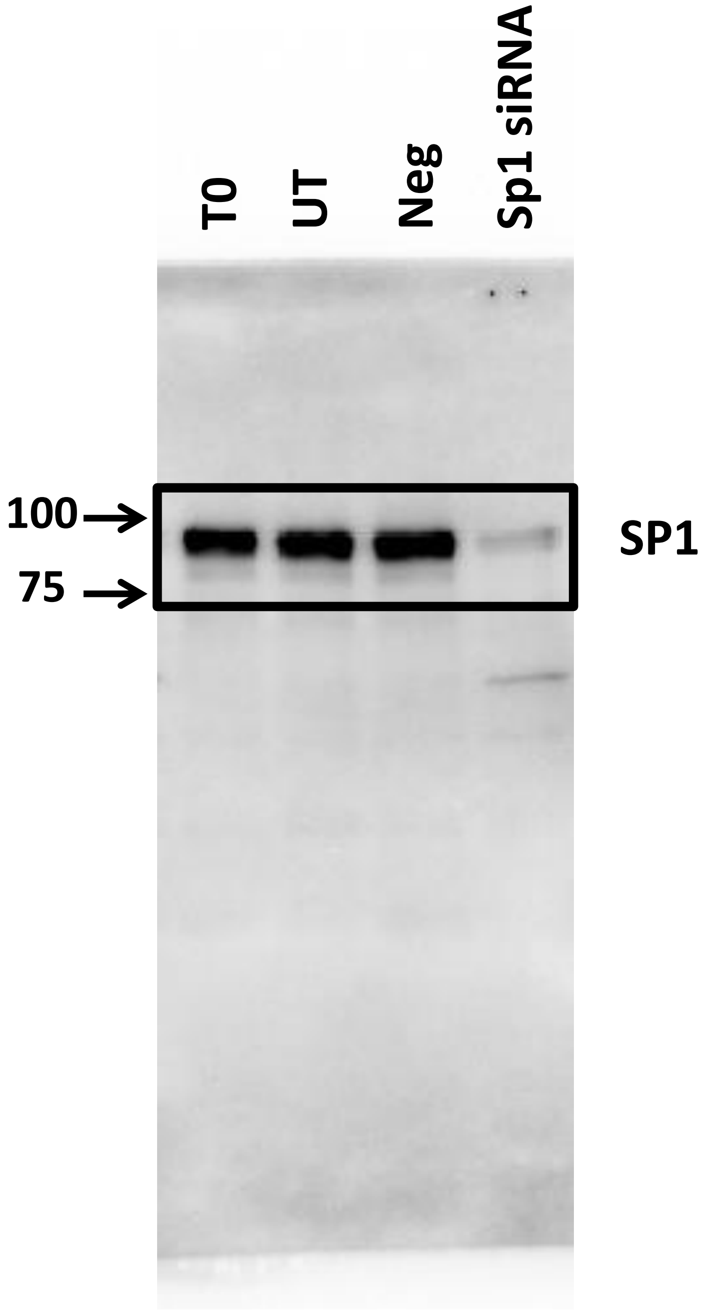

Supplementary Figure 3. Uncropped Western blot for SP1 represented in manuscript Figure 1E upper panel.

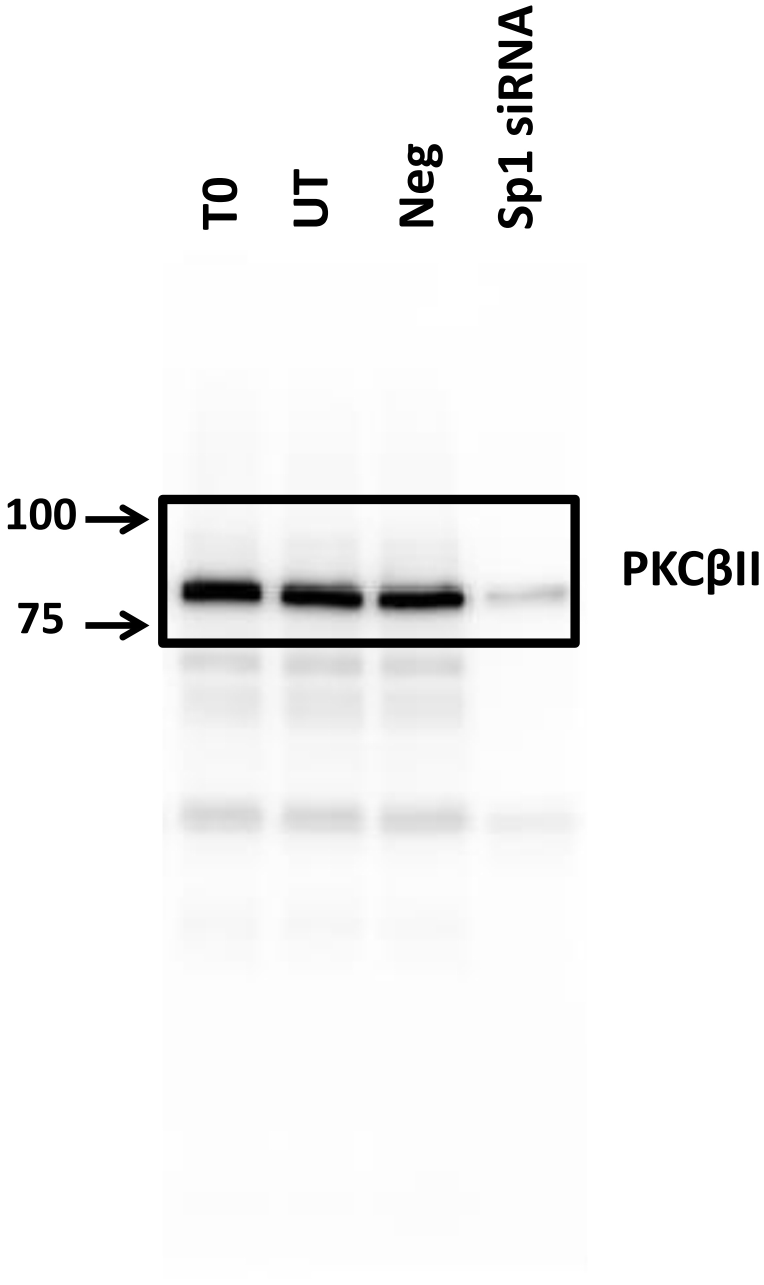

Supplementary Figure 4. Uncropped Western blot for PKCβII represented in manuscript Figure 1E middle panel.

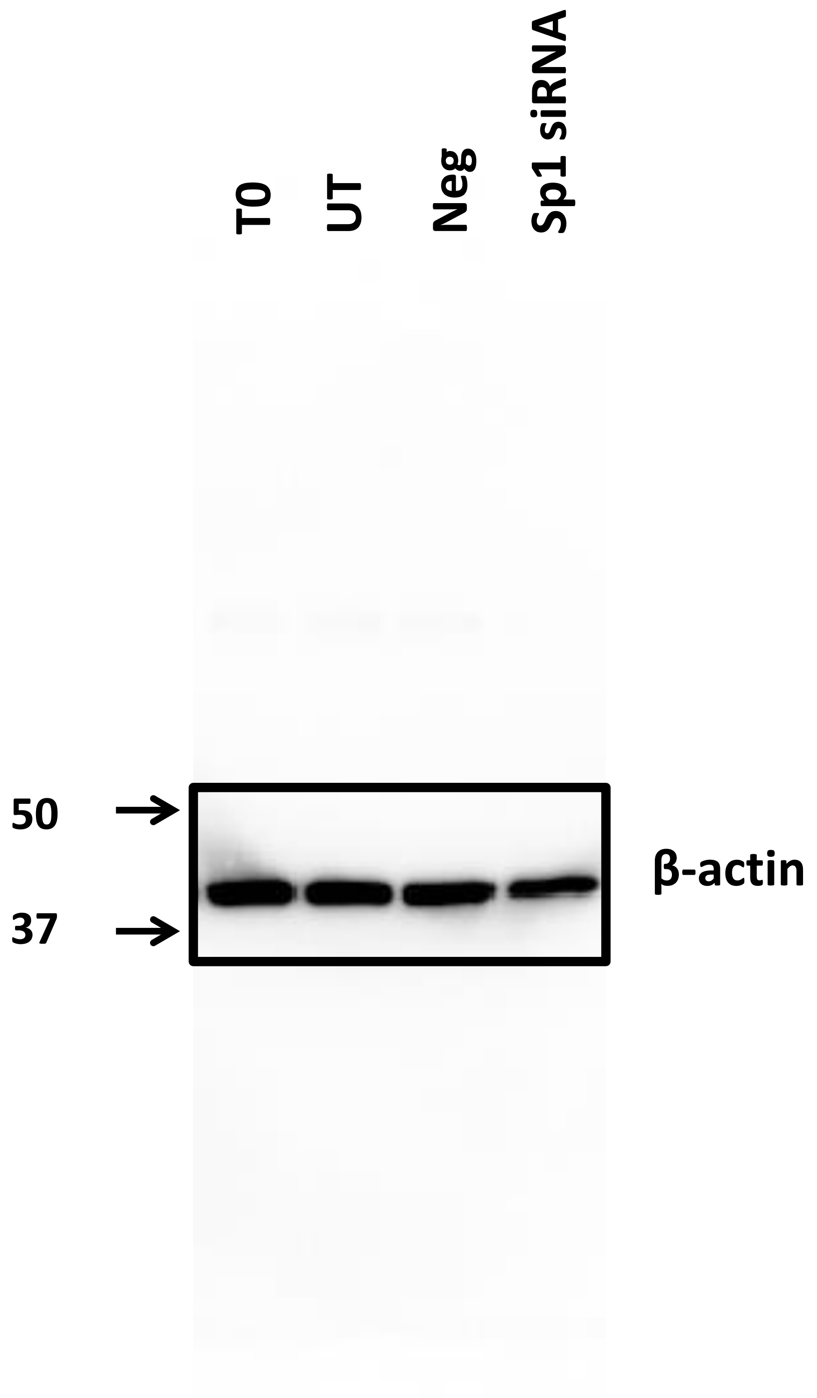

Supplementary Figure 5. Uncropped Western blot for β-actin represented in manuscript Figure 1E lower panel.
